# Decreased *ALKBH5*, *FTO*, and *YTHDF2* in Peripheral Blood Are as Risk Factors for Rheumatoid Arthritis

**DOI:** 10.1155/2020/5735279

**Published:** 2020-08-20

**Authors:** Qing Luo, Yujie Gao, Lu Zhang, Jiayue Rao, Yang Guo, Zikun Huang, Junming Li

**Affiliations:** ^1^Department of Clinical Laboratory, The First Affiliated Hospital of Nanchang University, Nanchang, China; ^2^Medical College, Nanchang University, Nanchang, Jiangxi 330006, China

## Abstract

ALKBH5 (alkylation repair homolog protein 5), FTO (fat mass and obesity-associated protein), and RNA N6-methyladenosine (m6A) demethylase, are essential for the m6A mRNA modification. YTHDF2 (YT521-B homology domains 2) called m6A “readers” can recognize m6A modification. As the key enzymes of m6A methylation modification, ALKBH5, FTO, and YTHDF2 have been implicated in many diseases. However, little is known about the role of ALKBH5, FTO, and YTHDF2 in rheumatoid arthritis (RA). We measured the mRNA expression of *ALKBH5*, *FTO*, and *YTHDF2* in RA patients and controls by quantitative real-time polymerase chain reaction, and the global m6A content was detected by an ELISA-like format. The mRNA expression of *ALKBH5*, *FTO*, and *YTHDF2* in RA patients was further analyzed to investigate its correlations with disease activity. And, multivariate analysis (logistic regression) was used to analyze the risk factors. The mRNA expression of *ALKBH5*, *FTO*, and *YTHDF2* in RA patients was significantly decreased compared to controls. The mRNA expression of *ALKBH5* was significantly increased in RA patients that received regular treatment. The mRNA expression of *FTO* was associated with disease activity score 28 (DAS28), complement 3 (C3), immunoglobulin G (IgG), and lymphocyte-to-monocyte ratio (LMR), some common markers for RA disease activity. The mRNA expression of *YTHDF2* was associated with RBC, L%, N%, NLR, and LMR. Furthermore, logistic regression analysis revealed that decreased expression of *ALKBH5*, *FTO*, and *YTHDF2* in peripheral blood was a risk factor for RA. Moreover, the peripheral blood global m6A content was significantly increased in patients with RA compared to CON, and increased m6A contents negatively correlated with decreased mRNA expression of FTO. In conclusion, this study firstly demonstrates the critical role of *ALKBH5*, *FTO*, and *YTHDF2* in RA, which provides novel insights into recognizing the pathogenesis of RA and a promising biomarker for RA.

## 1. Introduction

Rheumatoid arthritis (RA) is a chronic debilitating systemic autoimmune disease with permanent joint destruction, which is a highly disabling disease because of joint deformity and loss of function [1]. Due to its heterogeneity and multiplicity, the etiology of RA is still largely unknown [2]. Many studies have probed that the development of RA is attributed to genetic, infectious, environmental, and hormonal factors [3]. Accumulating studies have shown that dysregulation of the immune system, including abnormal activation T and B lymphocytes, neutrophils, mast cells, and macrophages, is involved in the mechanisms that drive the onset of RA [4, 5]. High levels of autoantibodies such as anticitrullinated protein antibodies (ACPA) generated by dysregulated B cells can cause lung destructions [6, 7]. Neutrophils and other inflammatory cells can arrive at sites of inflammation under stimulants from macrophages and mast cells, leading to joint injuries and deformity. Many current studies have probed the pathogenesis of RA, but the interference of different epigenetic alterations in RA is not fully understood.

In addition to DNA and histone modifications, epigenetic modifications of RNA have been proposed to be another layer of epigenetic regulation. Among RNA modifications, N6-methyladenosine (m6A) modification is the most prevalent in mammalian mRNA [8]. Despite m6A modification being first reported in early 1970s [9], its role and significance in RA are largely unknown. The key enzymes for m6A methylation modification primarily include m6A methyltransferase (writer), m6A demethylase (eraser), and m6A RNA-binding proteins (reader) [10]. Two well-known eraser enzymes, ALKBH5 (alkylation repair homolog protein 5) and FTO (fat mass and obesity-associated protein), are involved in mediating methylation reversal [11, 12]. It has been demonstrated that the role of ALKBH5 and FTO may alter in different tissues and cells. Evidences have found that ALKBH5 and FTO could promote cancer tumorigenesis [13, 14]. However, ALKBH5 and FTO have been reported as a tumor suppressor by inhibiting cancer progression [15, 16]. More interestingly, Huang et al. have found that ALKBH5 and FTO are associated with inflammation [17], and Lu et al. have shown that the expression of ALKBH5 and FTO mRNA in the liver of piglets was decreased after injection of LPS, which could take a significant role in hepatic injury during inflammation [18]. YTHDF2 (YT521-B homology domains 2) called m6A “readers” can recognize m6A modification [19], and YTHDF2 has been reported to regulate LPS-induced inflammatory response [20]. Thus, the role of ALKBH5, FTO, and YTHDF2 in RA, an autoimmune and inflammatory disease, still needs to be explored. In this study, we investigate the expression of ALKBH5, FTO, and YTHDF2 in RA and its relationship with disease activity.

## 2. Materials and Methods

### 2.1. Patient Variables

Patients (*n* = 79) who fulfilled the revised American College of Rheumatology (ACR) 2010 criteria for RA [21] were consecutively enrolled in the First Affiliated Hospital of Nanchang University between October 2018 and March 2019. Those RA patients accompanied by other autoimmune or inflammatory diseases, hormonal diseases, cancers, or mental disorders were excluded. All patients had new-onset RA and had not received corticosteroids or immunosuppressive drugs prior to recruitment. Then, 9 new-onset RA cases were administered therapeutic regimens with corticosteroids and immunosuppressive drugs for at least 15 days. The information on disease activity score 28 (DAS28), swollen joint count (SJC), tender joint count (TJC), patient visual analogue scale (VAS), erythrocyte sedimentation rate (ESR), C-reactive protein (CRP), anti-cyclic citrullinated peptide antibodies (Anti-CCP), rheumatoid factor (RF), white blood cell count (WBC), red blood cell count (RBC), hemoglobin, hematocrit (HCT), platelet count (PLT), lymphocyte count (L), lymphocyte percentage (L%), monocyte count (M), monocyte percentage (M%), neutrophil count (N), neutrophil percentage (N%), neutrophil-to-lymphocyte ratio (NLR), platelet-to-lymphocyte ratio (PLR), and lymphocyte-to-monocyte ratio (LMR) was collected. Health control (CON) (*n* = 61) without autoimmune or inflammatory diseases and who were also unrelated to patients with autoimmune diseases were randomly enrolled in the First Affiliated Hospital of Nanchang University between October 2018 and March 2019. All study protocols complied with the principles outlined in the Declaration of Helsinki and were approved by the Ethics Committee of the First Affiliated Hospital of Nanchang University (no. 019). All participants provided signed informed consent.

### 2.2. Collection of Peripheral Blood and Total RNA Extraction

Peripheral blood samples (2 ml) were collected into EDTA-2K containing tubes, and total RNA was extracted as soon as possible by using TRIzol reagent (Invitrogen, USA) according to the manufacturer's protocol. The concentration and integrity of the RNA was assessed by a NanoDrop ND-1000 spectrophotometer (Agilent Technologies, Inc.).

### 2.3. Reverse Transcription-Quantitative PCR (RT-qPCR) Analysis

Reverse transcription (RT) and quantitative PCR (qPCR) were carried out with the PrimeScript™ RT kit (Takara Bio Inc.) and SYBR Premix Ex Taq™ II (Takara Bio Inc.), respectively. RT-qPCR was performed on an ABI 7500 Real-Time PCR System (Applied Biosystems; Thermo Fisher Scientific, Inc.) with the following PCR thermocycler protocol: initial denaturation step at 95°C for 5 min, followed by 40 cycles of 95°C for 15 sec (denaturation), 60°C for 1 min (annealing and elongation), and 72°C for 2 min (final extension). GAPDH was used as an internal control. The primers used in RT-qPCR are listed in Table 1. The data were analyzed using the 2^-*ΔΔ*Ct^ method [22].

### 2.4. m6A RNA Methylation Analysis

Total RNA that was isolated from the peripheral blood of RA patients and CON was used to detect m6A RNA methylation by The EpiQuik™ m6A RNA Methylation Quantification Kit (Colorimetric) according to the manufacturer's protocol.

### 2.5. Statistical Analysis

Statistical analysis and graphic presentation were carried out with GraphPad Prism 5.0 (GraphPad Software, Inc.) and SPSS version 17.0 (SPSS Inc.). A Student's *t*-test was used between two groups where the samples passed the normality test; otherwise, the nonparametric Mann–Whitney test was used to analyze the data. The paired *t*-test was performed for the evaluation of changes in treatment. Spearman's method was used for correlation analysis. Multivariate regression analysis (logistic regression) was used to analyze the risk factors. *P* < 0.05 was considered to indicate statistically significant differences.

## 3. Results

### 3.1. Characteristics of Study Subjects

A total of 140 subjects were enrolled in the present study, including 79 patients with RA and 61 CON. RA patients were classified into screening and validation cohorts. The screening cohort included 20 RA patients and 20 CON. An independent cohort consisting of 59 RA patients and 41 CON was enrolled in the validation set for evaluation of abnormal genes. The characteristics of the study subjects are summarized in Table 2. There were no significant differences between RA patients and CON regarding age or sex. No correlation between the mRNA expression of *ALKBH5*, *FTO*, and *YTHDF2* in the peripheral blood, and age or sex was observed in RA or CON (data not shown).

### 3.2. Decreased mRNA Expression of ALKBH5, FTO, and YTHDF2 in the Peripheral Blood from RA Patients

**Figure 1:**
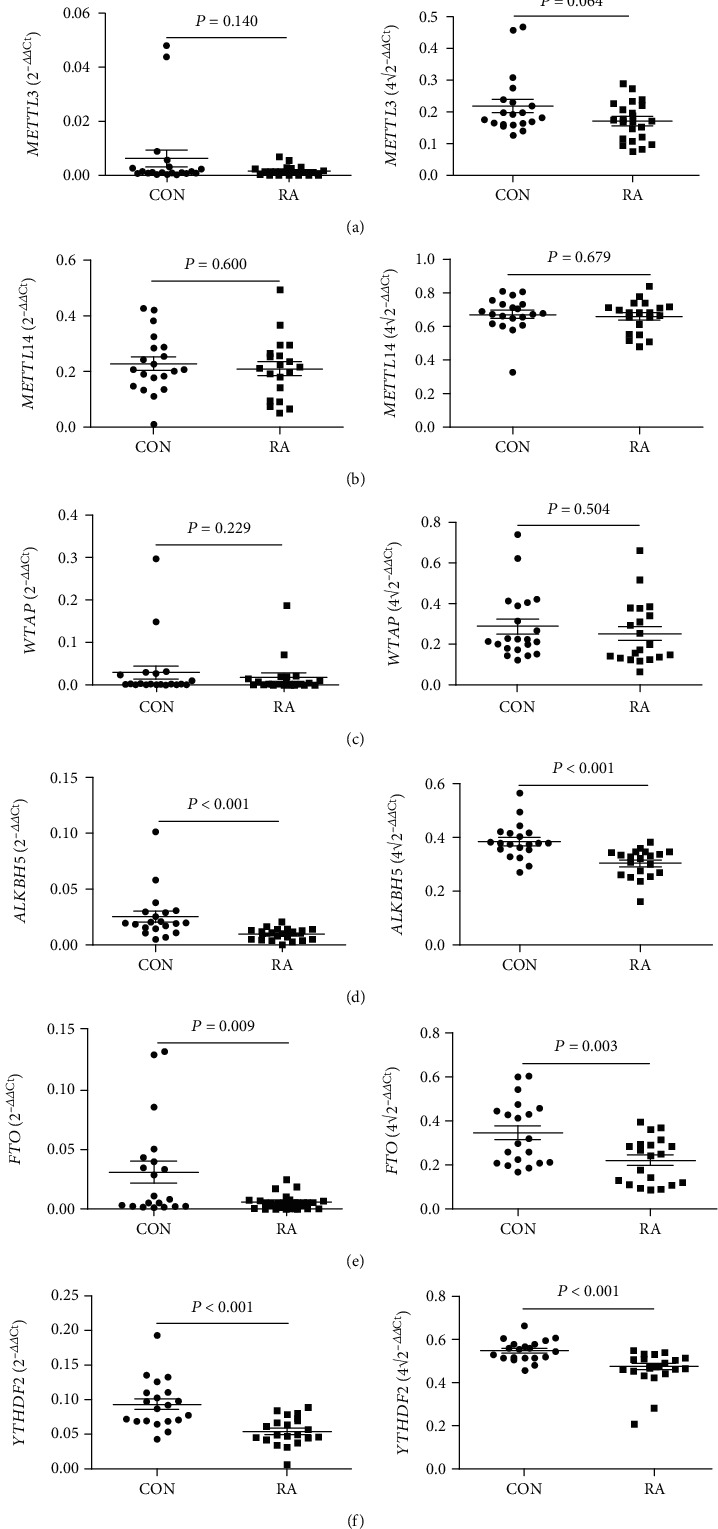
Reverse transcription-quantitative polymerase chain reaction (RT-qPCR) determined the mRNA expression of methyltransferase-like 3 (METTL3), methyltransferase-like 14 (METTL14), Wilms' tumor 1-associating protein (WTAP), A-ketoglutarate-dependent dioxygenase alkB homolog 5 (ALKBH5), fat mass and obesity-associated protein (FTO), and YT521-B homology domains 2 (YTHDF2) in the peripheral blood from 20 rheumatoid arthritis (RA) and 20 controls (CON). The average expression of METTL3 (a), METTL14 (b), and WTAP (c) did not show any remarkable differences between patients with RA and the CON. The average expression of ALKBH5 (d), FTO (e), and YTHDF2 (f) in patients with RA was significantly decreased than those in CON.

**Figure 2:**
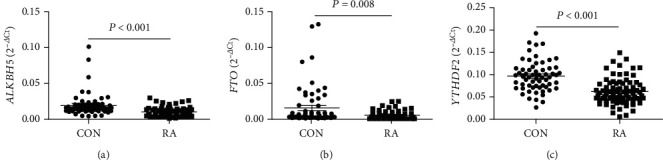
Validation the mRNA expression of A-ketoglutarate-dependent dioxygenase alkB homolog 5 (ALKBH5), fat mass and obesity-associated protein (FTO), and YT521-B homology domains 2 (YTHDF2) in the peripheral blood from rheumatoid arthritis (RA). The average expression of ALKBH5 (a), FTO (b), and YTHDF2 (c) in patients with RA was significantly decreased than those in controls (CON).

To identify the mRNA expression of *METTL3*, *METTL14*, *WTAP*, *ALKBH5*, *FTO*, and *YTHDF2* in the peripheral blood from RA patients and CON, we used qRT-PCR to assess these gene expressions in screening testing set consisting of 20 RA patients and 20 CON. Data showed that although the mRNA expression of *METTL3*, *METTL14*, and *WTAP* was unchanged (all *P* > 0.050) (Figures 1(a)–1(c)), the mRNA expression of *ALKBH5*, *FTO*, and *YTHDF2* in the peripheral blood was significantly decreased in RA patients compared to CON (all *P* < 0.050) (Figures 1(d)–1(f)). Subsequently, an independent validation testing set consisting of 59 RA patients and 41 CON was enrolled and determined for their *ALKBH5*, *FTO*, and *YTHDF2* levels. From the data of all the RA patients and CON, the mRNA expression of *ALKBH5*, *FTO*, and *YTHDF2* in the peripheral blood from 79 RA patients was significantly lower compared to 61 CON (all *P* < 0.050) (Figures 2(a)–2(c)).

### 3.3. Correlation of ALKBH5, FTO, and YTHDF2 Expression in the Peripheral Blood with Clinical Features of RA

**Figure 3:**
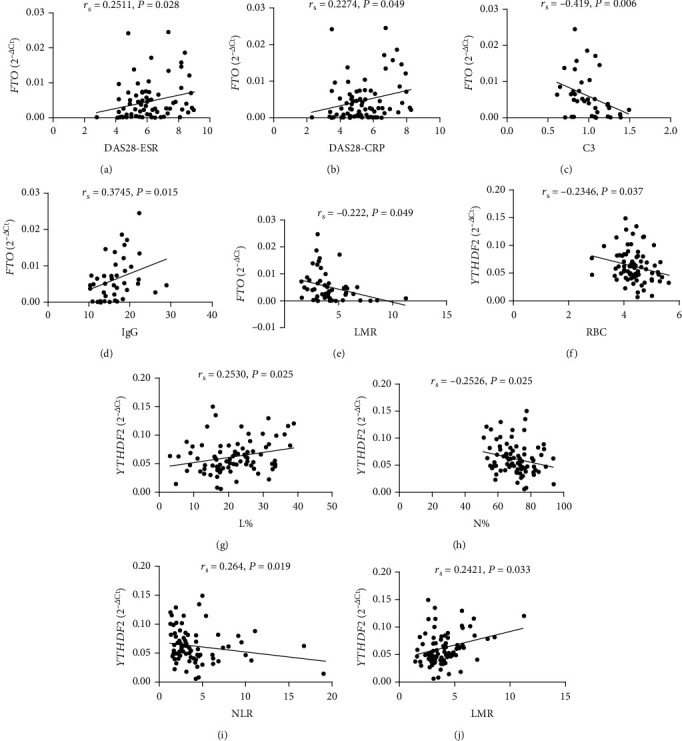
Correlation of the mRNA expression of fat mass and obesity-associated protein (*FTO*) and YT521-B homology domains 2 (*YTHDF2*) in rheumatoid arthritis (RA) with clinical features. The mRNA expression of *FTO* in RA positively correlated with disease activity score (DAS28) (a, b) and immunoglobulin G (IgG) (d). The mRNA expression of FTO in RA negatively correlated with complement 3 (C3) (c) and lymphocyte-to-monocyte ratio (LMR) (e). The mRNA expression of *YTHDF2* in RA negatively correlated with red blood cell count (RBC) (f), neutrophil percentage (N%) (h), and neutrophil-to-lymphocyte ratio (NLR) (i). The mRNA expression of *YTHDF2* in RA positively correlated with lymphocyte percentage (L%) (g) and LMR (j).

To determine whether the mRNA expression of peripheral blood *ALKBH5*, *FTO*, and *YTHDF2* from RA patients could reflect the activity of the disease, clinical features including DAS28-ESR, DAS28-CRP, VAS, SJC, TJC, Anti-CCP, RF, ESR, CRP, C3, C4, IgG, WBC, RBC, HGB, HCT, PLT, L, L%, M, M%, N, N%, PLR, NLR, LMR, and duration were collected, and analysis was performed to assess the correlation between the clinical features of RA and the mRNA expression of the peripheral blood *ALKBH5*, *FTO*, and *YTHDF2*. As shown in Figure 3, the mRNA expression of peripheral blood *FTO* correlated with DAS28-ESR, DAS28-CRP, C3, IgG, LMR, and the mRNA expression of the peripheral blood *YTHDF2* correlated with RBC, L%, N%, NLR, and LMR. However, no correlation was found between these clinical features of RA and the mRNA expression of peripheral blood *ALKBH5* (data no shown).

**Figure 4:**
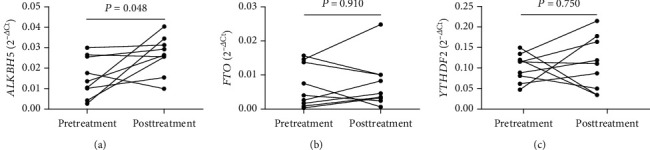
Correlation of the mRNA expression of A-ketoglutarate-dependent dioxygenase alkB homolog 5 (*ALKBH5*), fat mass and obesity-associated protein (*FTO*), and YT521-B homology domains 2 (*YTHDF2*) in rheumatoid arthritis (RA) with treatment. The mRNA expression of *ALKBH5* (a) was significantly increased in RA patients that received regular treatment. The mRNA expression of *FTO* (b) and *YTHDF2* (c) did not show any remarkable differences between pretreatment and posttreatment.

Subsequently, the mRNA expression of peripheral blood *ALKBH5*, *FTO*, and *YTHDF2* was detected in 9 new-onset RA cases pre- and posttreatment. Notably, the mRNA expression of peripheral blood *ALKBH5* in 7 of the RA patients increased following the treatment when compared with those prior to treatment, and 2 RA patients had decreased mRNA expression of peripheral blood ALKBH5. As shown in Figure 4, after treatment, there was a significant difference; however, there was no difference between pre- and posttreatment levels in the mRNA expression of the peripheral blood *FTO* and *YTHDF2*.

### 3.4. The Expressions of ALKBH5, FTO, and YTHDF2 in the Peripheral Blood Were Risk Factors for RA

The aforementioned results (Figures 3 and 4) demonstrate that the decreased mRNA expression of *ALKBH*5 in the peripheral blood correlated with treatment, and the decreased mRNA expression of *FTO* and *YTHDF*2 in the peripheral blood correlated with disease activity. Thus, we investigated whether *ALKBH*5, *FTO*, and *YTHDF*2 were risk factors for RA using the “enter method” of multivariate logistic regression. As shown in Table 3, the equations about the levels of peripheral blood *ALKBH*5, *FTO*, and *YTHDF*2 were obtained, *Y* = −87.526 × 1 (*ALKBH*5) − 54.550 × 2(*FTO*) − 23.192 × 3(*YTHDF*2) + 3.592. Importantly, multivariate regression analysis revealed that decreased mRNA expressions of *ALKBH*5, *FTO*, and *YTHDF*2 in peripheral blood were risk factors for RA (*P* = 0.019; *P* = 0.029; and *P* < 0.001), suggesting *ALKBH*5, *FTO*, and *YTHDF*2 may play prominent pathogenic roles in the development and progression of RA.

### 3.5. The Increased Global m6A Contents Negatively Correlated with Decreased mRNA Expression of FTO

**Figure 5:**
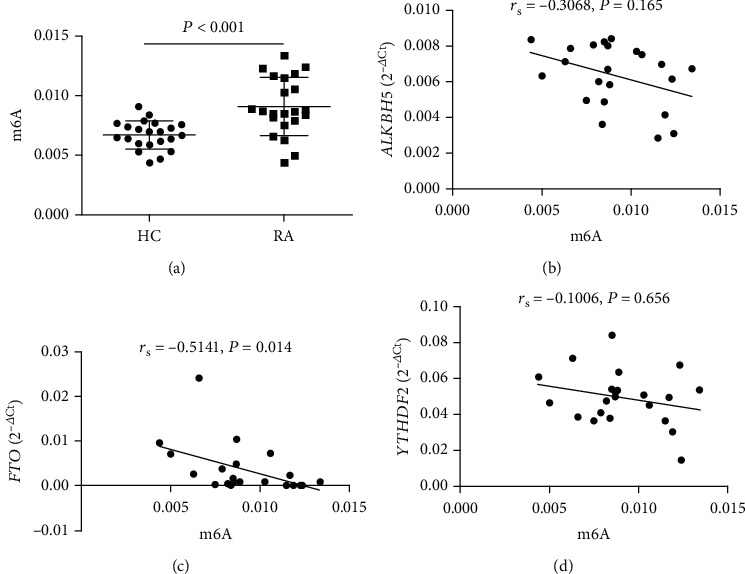
The correlations between the global m6A content and the mRNA expression of A-ketoglutarate-dependent dioxygenase alkB homolog 5 (*ALKBH5*), fat mass and obesity-associated protein (*FTO*), and YT521-B homology domains 2 (*YTHDF2*) in the peripheral blood from rheumatoid arthritis (RA). The peripheral blood global m6A content was significantly increased in patients with RA compared to controls (CON) (a). The increased m6A contents negatively correlated with decreased mRNA expression of *FTO* (c) and did not correlated with decreased mRNA expression of *ALKBH5* (b) and *YTHDF2* (d).

Multivariate regression analysis showed that the decreased mRNA expressions of m6A demethylase (*ALKBH5*, *FTO*) and m6A RNA-binding proteins (*YTHDF2*) were all risk factors for RA. Thus, we detected the global m6A content in peripheral blood and investigated the correlations between the global m6A content and the mRNA expression of *ALKBH5*, *FTO*, and *YTHDF2* in the peripheral blood. As shown in Figure 5, the peripheral blood global m6A content was significantly increased in patients with RA compared to CON (*P* < 0.001), and the increased m6A contents negatively correlated with decreased mRNA expression of *FTO* (*r*_s_ = −0.5141, *P* = 0.014).

## 4. Discussion

m6A is a methylation at the N6 position of adenosine, which is regarded as the most abundant epitranscriptomic modification of mRNA in eukaryotic cells [23]. Abnormal m6A modification may lead to dysfunction of RNA, which can further trigger some diseases in both animals and humans [14, 24]. There are several key genes involved in m6A methylation modification, primarily including METTL3, METTL14, WTAP, FTO, ALKBH5, and YTHDF2 [25, 26]. Given the effects of these key genes involving in m6A methylation modification on the pathogenesis of many disease [14, 24], we firstly detected the expression of m6A methylation-associated genes (*METTL3*, *METTL14*, *WTAP*, *FTO*, *ALKBH5*, and *YTHDF2*) in the peripheral blood from RA patients and showed that the expression of *ALKBH5*, *FTO*, and *YTHDF2* in the peripheral blood from RA patients was significantly lower than CON, while the expression of *METTL3*, *METTL14*, and *WTAP* was unchanged. The reason of downregulated m6A regulators in RA compared with CON may be the environmental, infectious, and genetic factor, as well as some RA risk factors (unbalance of adaptive and innate immune). Recently, Wang and colleagues have investigated the expression of METTL3, FTO, ALKBH5, METTL14, and YTHDF2 in peripheral blood mononuclear cell from RA patients and reported conflicting results in which only METTL3 was obviously upregulated in RA [27]. The reasons for these outcomes are probably due to differences in cell type and the disease duration.

Thus, we investigated whether the expression of peripheral blood *ALKBH5*, *FTO*, and *YTHDF2* from RA patients could reflect the activity of the disease and inflammatory response. We found that the expression of the peripheral blood *ALKBH5* increased following the treatment when compared with those prior to treatment. We showed that the expression of peripheral blood *FTO* correlated with DAS28-ESR, DAS28-CRP, TJC, C3, IgG, L, PLR, and LMR, which indicated the activity of the disease. In addition, the expression of peripheral blood *YTHDF2* correlated with RBC, L%, N%, NLR, and LMR. These results indicated that m6A demethylase *ALKBH5*, *FTO*, and *YTHDF2* were associated with disease activity and inflammatory response. Evidences from other disease indicated that *ALKBH5* and *FTO* might be used as prognostic markers. Yang and colleagues showed that *ALKBH5* was an independent prognostic indicator of overall survival and disease-free survival in colon cancer patients [28]. Xu and colleagues have found that the expression of *FTO* was positively correlated with TNM stage, and the Kaplan-Meier analysis showed that high *FTO* expression was significantly associated with poor prognosis in GC patients [29]. Moreover, evidences have indicated that FTO and YTHDF2 are associated with inflammation [17, 20]. Our results also showed the expression of peripheral blood *ALKBH5*, *FTO*, and *YTHDF2* may use as indicator of activity and inflammatory response.

The expression of peripheral blood *ALKBH5*, *FTO*, and *YTHDF2* in peripheral blood was significantly decreased in RA patients, and the expressions of peripheral blood *ALKBH5*, *FTO*, and *YTHDF2* were associated with disease activity and inflammation. Thus, we explored whether the expressions of the peripheral blood *ALKBH5*, *FTO*, and *YTHDF2* were risk factors for RA. In agreement with previous results, a logistic regression analysis revealed that decreased expressions of *ALKBH5*, *FTO*, and *YTHDF2* in peripheral blood were risk factors for RA. Our results suggested that *ALKBH5*, *FTO*, and *YTHDF2* may play prominent pathogenic roles in the development and progression of RA.

As we have known, FTO and ALKBH5 are involved in mediating methylation reversal. Thus, we detected global m6A content in the peripheral blood from RA patients and CON. And, the results showed that the peripheral blood global m6A content was significantly increased in patients with RA compared to CON. Moreover, we investigated the correlations between the global m6A content and the mRNA expression of *ALKBH5*, *FTO*, and *YTHDF2* in the peripheral blood, and we found that the increased m6A contents negatively correlated with decreased mRNA expression of *FTO.* Although FTO was decreased and global m6A contents were increased, we did not know which mRNA that plays an important role in RA was methylated. In addition, as the most prevalent modification of RNA, m6A methylation is a double-edged sword for many diseases, over m6A modification of certain genes could lead to alterations of mRNA behavior and expression, resulting in the acceleration of disease development, whereas the lack of m6A modification on other genes may also lead to disease progression [30]. Thus, It is possible that the downregulated m6A regulators in RA positively correlated with indicators of disease progression.

In conclusion, the current study firstly measured the expression of *METTL3*, *METTL14*, *WTAP*, *ALKBH5*, *FTO*, and *YTHDF2* in the peripheral blood from RA patients and showed that dysregulated *ALKBH5* and *FTO* were associated with RA. In addition, we found that the expression of peripheral blood *ALKBH5* and *FTO* was associated with autoantibody production and disease activity. The findings in this study are useful for understanding RA pathogenesis and exploring novel biomarkers for RA diagnosis and treatment.

## Figures and Tables

**Table 1:** The amplification primers sequences.

Gene name	Sequence (5′-3′)
METTL3	F: AAGCTGCACTTCAGACGAAT
	R: GGAATCACCTCCGACACTC
METTL14	F: AGAAACTTGCAGGGCTTCCT
	R: TCTTCTTCATATGGCAAATTTTCTT
WTAP	F: GGCGAAGTGTCGAATGCT
	R: CCAACTGCTGGCGTGTCT
ALKBH5	F: CCCGAGGGCTTCGTCAACA
	R: CGACACCCGAATAGGCTTGA
FTO	F: TGGGTTCATCCTACAACGG
	R: CCTCTTCAGGGCCTTCAC
YTHDF2	F: GGCAGCACTGAAGTTGGG
	R: CTATTGGAAGCCACGATGTTA
GAPDH	F: TGCACCACCAACTGCTTAGC
	R: GGCATGGACTGTGGTCATGAG

Methyltransferase-like 3 (METTL3), methyltransferase-like 14 (METTL14), Wilms' tumor 1-associating protein (WTAP), A-ketoglutarate-dependent dioxygenase alkB homolog 5 (ALKBH5), fat mass and obesity-associated protein (FTO), and YT521-B homology domains 2 (YTHDF2).

**Table 2:** Clinical details of the patients with RA and HC.

Clinical characteristic	RA	CON
Number of subjects	79	61
Sex, male/female	15/64	12/49
Age, years	51.35 ± 12.26	48.66 ± 14.09
Duration, day	1439.82 ± 2246.33	
DAS28-ESR	6.12 ± 1.38	
DAS28-CRP	5.43 ± 1.31	
SJC	11.26 ± 7.10	
TJC	13.64 ± 6.93	
VAS	54.35 ± 28.52	
RF, IU/ml	507.39 ± 613.34	
Anti-CCP, RU/ml	614.87 ± 772.44	
ESR, mm/h	57.34 ± 33.78	
CRP, mg/l	35.03 ± 37.63	
IgG, g/l	16.54 ± 4.12	
C3, g/l	0.98 ± 0.21	
C4, g/l	0.23 ± 0.09	
WBC, 10^9^/l	7.90 ± 2.40∗	5.69 ± 0.95
RBC, 10^12^/l	4.37 ± 0.51∗	4.55 ± 0.36
HGB, g/l	122.68 ± 18.37∗	136.98 ± 11.08
HCT, l/l	0.38 ± 0.05∗	0.41 ± 0.03
PLT, 10^9^/l	334.92 ± 135.74∗	236.51 ± 41.34
L, 10^9^/l	1.61 ± 0.56∗	1.97 ± 0.38
L, %	21.42 ± 8.26∗	35.13 ± 6.94
M, 10^9^/l	0.43 ± 0.18∗	0.35 ± 0.08
M, %	5.71 ± 2.07	6.22 ± 1.41
N, 10^9^/l	5.70 ± 2.26∗	3.24 ± 0.80
N, %	70.67 ± 9.77∗	56.10 ± 7.01
PLR	242.55 ± 178.36∗	123.65 ± 28.00
NLR	4.23 ± 3.12∗	1.72 ± 0.58
LMR	4.56 ± 4.97∗	5.97 ± 1.63

∗*P* < 0.05 RA compared to CON. Anti-cyclic citrullinated peptide antibodies (Anti-CCP), health control (CON), C-reactive protein (CRP), disease activity score (DAS28), erythrocyte sedimentation rate (ESR), hematocrit (HCT), hemoglobin (HGB), lymphocyte count (L), lymphocyte percentage (L%), lymphocyte-to-monocyte ratio (LMR), monocyte count (M), monocyte percentage (M%), neutrophils count (N), neutrophils percentage (N%), neutrophil-to-lymphocyte ratio (NLR), platelet-to-lymphocyte ratio (PLR), platelet count (PLT), tender joint count (TJC), rheumatoid arthritis (RA), red blood cell count (RBC), rheumatoid factors (RF), swollen joint count (SJC), Visual Analogue Scale (VAS), and white blood cell count (WBC).

**Table 3:** Clinical details of the patients with RA and HC.

	*B*	SE	Wald	*df*	*P*	Exp (*s*)
*ALKBH5*	-87.526	37.379	5.483	1	0.019	0.000
*FTO*	-54.550	24.954	4.779	1	0.029	0.000
*YTHDF2*	-23.192	8.186	8.026	1	0.005	0.000
Constant	3.592	0.656	29.974	1	0.000	36.320

A-ketoglutarate-dependent dioxygenase alkB homolog 5 (*ALKBH5*), fat mass and obesity-associated protein (*FTO*), and YT521-B homology domains 2 (*YTHDF2*).

## Data Availability

The data used to support the findings of this study are available from the corresponding authors upon request.
